# Family experience in facilitating adolescents during self-identity development in ex-localization in Indonesia

**DOI:** 10.1186/s12912-019-0358-7

**Published:** 2019-08-16

**Authors:** Uswatun Hasanah, Herni Susanti, Ria Utami Panjaitan

**Affiliations:** 1 Magister of Nursing Science, Specialty in Mental Health Nursing, Faculty of Nursing, Universitas Indonesia, Depok, Indonesia; 2 Department of Mental Health Nursing, Faculty of Nursing, Universitas Indonesia, Depok, Indonesia

**Keywords:** Self-identity, Localization, Family experience, Adolescents

## Abstract

**Background:**

Self-identity is a personal reflection that is consistent and covers various individual aspects, such as job/career, spirituality, relations, intellectuality, sexuality, culture, interests, personality, and physical identity. The increasing level of juvenile delinquency worldwide, including in Indonesia, is a manifestation of unsuccessful identity development in adolescents. Self-identity development is inseparable from family influence. This study aimed to explore the experiences of families in facilitating their adolescents during self-identity development while living in ex-localization.

**Methods:**

This study used a descriptive qualitative design and involved 12 participants. Data were collected through in-depth interviews and analyzed using thematic analysis.

**Results:**

This study resulted in five themes: the identity achievement of adolescents living in ex-localization is similar to that of adolescents in general; the domination of external barriers during identity achievement; ex-localization as a stressor; families’ efforts to facilitate their adolescents during identity achievement; and family expectations for the future.

**Conclucions:**

This study highlights the importance of improving family awareness of adolescents’ identity achievement when living in ex-localization with the help of nursing mental health professionals.

## Background

Based on the population projection data in 2015, there were 66 million adolescents in Indonesia, representing approximately 25% of the country’s total population [1] Meanwhile, the number of adolescents living in the province of the research site is 426.786; and 250 of them are living in ex-localization Dupak Bangun Sari, Surabaya city [2].

Adolescent development is characterized by identity achievement, which is indicated by the ability of the individual to understand his or her roles in the community, select a job he or she desires. The adolescent is also expected to behave in accordance with his or her religious values, make decisions without involving other people, gain academic achievements, set goals, engage in positive hobbies, and socialize with his/her surroundings. According to Erikson, adolescents must overcome self-identity crises and identity confusion [3]. Adolescents who are able to complete these developmental tasks would have positive self-identities. On the contrary, those who cannot complete these developmental tasks would experience identity confusion [3]. Identity confusion is indicated by their inability to value their roles in the community, unstable personalities, a lack of goals, hobbies, or plans for their future, a bad attitude, and no interest in daily activities.

Adolescents living in ex-localization are expected to complete their developmental tasks and acquire positive self-identities. However, many of them continue to engage in socially unacceptable activities, such as theft, smoking in coffee shops, playing billiards at school time and at night, spending time in karaoke bars, displaying strong affection to a member of the opposite sex in public, and dressing sexually [4]. On the other hand, some adolescents in ex-localization areas exhibit more constructive behaviors and habits, such as participating in group Quranic recitations and social activities in local mosques and youth organizations [4]. All teenagers, including those living in ex-localization, show diverse performance during their developmental task achievement. Support from the adolescents’ families is expected during this process [5].

Family has a major influence on adolescents in terms of limiting risk-taking behavior such as unsafe premarital sex and drug use [6]. If these behaviors are uncontrolled, the adolescents could experience a so-called identity crisis [5]. Putra emphasizes that interpersonal communication between parents and children is important to help youths avoid sex before marriage [6] Further, a Southwest Wisconsin Youth Survey revealed that parental monitoring is a powerful tool for preventing adolescents from engaging in risk-taking behaviors [7].

Adolescent identity development is also influenced by parenting style [8]. Teenagers who do not experience abuse within their family achieve clearer identity than those who do [9]. Some researchers find that a positive parenting style lead to positive behaviors in their children, and the opposite is also true [10]. It is argued that parental background (family) is one of many factors that influence adolescents during their self-identity development [5].

In addition to family, the community and individuals living locally in localization influence self-identity development in adolescents. This localization may have adverse effects on people and their surroundings, including adolescents. Such adverse effects include disturbances to households and family life, personality issues, the spread of sexually transmitted diseases, and sexual crimes involving teenagers and the people around them [11]. Clearly, such conditions would hamper self-identity development in adolescents.

Some researchers found that the common localization problems faced by teenagers in Gambilagu, Semarang, Indonesia are fighting, having no interactions with other teenagers outside the local area, and engaging in other negative behaviors [4]. Conversely, the positive interactions between family life risk and protective factors with regard to localization in Dupak Bangunsari in Surabaya, Indonesia have been linked to resilient behaviors [11].

Based on this, the question raised in this study is how the family experience facilitates adolescents in developing their self-identities in ex-localization. The novelty in this study is exploring self-identity developmental tasks in more depth from a family point of view, specifically the adolescents’ parents. In addition, this study was conducted with families and teenagers living in ex-localization.

## Method

A descriptive qualitative method was employed in this study. A qualitative research design using a descriptive approach should yield results that adequately reflect the research data [12]. This research design focused on fact-finding regarding a social phenomenon, to understand human behaviors based on the participants’ perspectives. The participant recruitment was assisted by gatekeepers (i.e. local community leaders) who provided information about potential participants. The participants were then selected using a purposive sampling method. Based on this consideration, the inclusion criteria were set as follows: (a) family living in ex-localization in Dupak Bangunsari, Surabaya, Indonesia and had adolescents aged 12–18 years who were not yet married, (b) having interaction with the adolescents > 2 h per day, and (c) the participants were the parents of those adolescents. The selection process resulted in 12 candidates to become participants, who had adolescents with age 12–18 years old.

The main researcher (i.e. first author) collected the data through in-depth interviews that were conducted in each participant’s home. Prior to interviewing, the main researcher contacted prospective participants one by one either by phone or directly visit their homes. Then, the researcher gives an explanation sheet to the prospective participants.

At this stage, the main researcher gave opportunity to the prospective participants to think for 24 h. After the time given to think over, the researcher contacted the prospective participant to ask for the decision while fixed the meeting time for the interview. In addition to providing explanations to prospective participants, the mzin researcher had practiced under the supervision of her supervisors (i.e. the second and third authors) who had significant experiences in qualitative inquiries.

Data processing and analysis employed thematic analysis to facilitate the emergence of themes inductively [13]. During the analysis process, all research members involved in the theme generation and interpretation. This study passed ethical review by the Ethics Committee of Nursing Department, Universitas Indonesia, which referred to the following ethical principles: *Beneficence*, *Respect for Human Dignity*, and *Justice.* The credibility of the investigation was enhanced through the peer debriefing by working together with supervisors who posed more neutral views of the research. The supervisors examined and provided feedback of the main author’s transcripts, interpretation and final results. In addition, the dependability of the study was accomplished by undertaking an audit trail when the researcher discussed with her supervisors surrounding the progress of data collection and analysis. Regular meetings with the supervisors were helpful in revealing the main researcher’s bias, assumptions, and data misinterpretation.

## Results

### They are same with teenagers in general

According to the participants there were no difference between the teenagers living in ex-localization and their peers in general with regard to physical change, appearance change, behavioral change, the ability to build relationships with members of the opposite sex, and choosing activities based on their own interests. The following statements reflect the participants’ observations:

“Well, the change was just as usual, like breast growth, and also menstruation.” (P9).

“(she) asks for something decent (clothes). And then, asks for nice sandal, ask for good hair style.” (P1).

“Well, the change like complaining, which is normal as he already grew up.” (P3).

### External barriers were dominant during identity achievement

The family barriers to facilitating the adolescents’ identity achievement in ex-localization were dominated by external factors such as environment condition, peer influence, and social stigma:

“Well, anything, like drugs, premarital sex, exist.” (P12).

“He started to smoke because of friends.” (P11).

“People asked what’s he gonna be if living in Bangunsari” (P7).

There were also one internal factor that was a barrier during the adolescents’ identity achievement: financial issues. One participant noted this problem:

“Actually, he’s going to be graduated, and he wants to continue to college, but we have no money, so...” (P6).

The external and internal barriers felt by the families that have teenagers in ex-localization environment are directly proportional to social phenomena occurring in society. The phenomenon showed that many teenagers are unable to achieve developmental tasks such as completing education, getting decent work, and having inability to value a role in society. It happens because of the influence of interaction with peers, embarrassment caused by stigma given by others outside the neighborhood, as well as insufficient economic factors.

The economic factors have a distinct impact on teenagers. Inadequate family economic conditions got the teenagers to work as breadwinners. This condition forced the role of teenagers who should take education, join organizational activities or extracurricular to be change as breadwinners.

The phenomenon above showed that there are teenagers who cannot achieve the task of development, whereas it does not make barriers for other families to have their teenagers can achieve the task of development well. Seven of the twelve participants indicated that they were able to assist their teenagers, so that they could perform their role in achieving the task of development well.

### Ex-localization as a stressor

The environment in which they lived had psychological impacts on the families and teenagers. All the participants expressed similar psychological responses to the ex-localization enviroment, including fear, worry, and anxiety, and one of the participants even expressed sad feelings while crying:

“We’re afraid he’s going to follow [bad behaviors]” (P11).

“if it is so intimate, so intimate with them [commercial sex worker], they are invited in room to watch TV. As long as I’m still worried” (P2).

“yah, afraid of this place [ex-localization] neighborhood.” (P12).

“it is really sad, i feel like i want to cry with tears full with blood.” (P8).

The existence of ex-localization environment becomes a stressor for families with teenagers. The researchers observed that some parents do not give permission to their teenagers to get along with other teenagers inside ex-localization environment as they feel worried about their teenagers will have bad behaviors and habits.

Five of the twelve participants said that it was better for their teenagers to do activities outside ex-localization environment than to interact with other teenagers in the neighbourhood.

### Families’ efforts to facilitate their adolescents during identity achievement

Monitoring, advising, avoiding violence, and instilling religious value were the efforts the families made to facilitate their adolescents during identity achievement in ex-localization. Some of the participants described their efforts:

“Well, there are a lot here. But my child, I keep my eyes on him always. Anywhere, I’ll be watching.” (P1).

“Well, I just keep trying, keep advising.” (P11).

“If we scolding him too bad, we’re worried he’s gonna be worse.” (P11).

“For me, religion is number one, I emphasize it, thing that’s going to protect us is religion.” (P8).

### Family expectation for the future

In facilitating their adolescents’ identity achievement, the families had good expectations of their children and government:

“I want, someday, for my child to at least have a bachelor degree.” (P6).

“If possible, give them positive activities, like Qur’an recitation, and preach.” (P3).

## Discussion

### Identity achievement of adolescents living in ex-localization is similar to that of adolescents in general

In this study, the families expressed that the identity achievements of their children in terms of changes were similar to the achievements of adolescents in general. These changes included physical, appearance, and behavioral changes, the ability to build relationships with members of the opposite sex, and the ability to choose activities based on their own interests. Brownlee [14] states that development in early adolescence is characterized by rapid physical changes including height, maturity of reproductive organs, secondary sex characteristics, increased muscle strength, and weight [15, 16]. The biological changes are signs of maturity, such as the functioning of reproductive organs (menstruation in females and nocturnal emissions in males), as the adolescents are moving toward adulthood. Interpretations of identity achievement are influenced by the families’ perceptions, educational backgrounds, and the closeness between the families and the adolescents [5]. The process of identity formation in adolescents is influenced by various factors, one of which is the background of the parents [5].

### External barriers were dominant during identity achievement

The barriers that were faced by the families in facilitating their adolescents were mostly due to external factors such as environmental conditions, peer influence, and social stigma. The participants expresed that a bad environment would hamper the adolescents’ identity development in many ways. There is a place where still has cafes in which still operate and serve prostitution transaction, shops which provide alcohol, free-drug trafficking, and free-sex which may cause parents more vigilant on giving permission to their adolescents to interact around their environment.

In the general environment without prostitution activity, parents may not worry so that they can give chance to their adolescents to explore and interact with the local people including peers. Also, there is no social stigmatization postulates that the society, especially adolescents, have bad behaviors caused by bad environment. This was asserted by most of participants of this study in that they were more comfortable to give permission to their adolescents to interact with and visit their school mates as long as it located in outside ex-localization areas. Clearly, these two conditions give the difference of how families in ex-localization environment and those who live outside ex-localization in facilitating their adolescents to achieve self-identity.

In addition, juvenile deliquency has been the most common problem in this ex-localization. This deliquency is the manifestation of an identity crisis, where the juvenile is unable to adapt to the existing stressors so responds in a maladaptive way, often reflected in destructive behaviors. These behaviors are including smoking, inappropriate diet, lack of physical activity, drug and alcohol abuse, premarital sex, violence, suicide, murder, and motor vehicle accidents [17].

Friends of the same age also contributed to barriers to the adolescents’ identity achievement. The participants in the study stated that both the positive and negative behaviors of their teenagers were mostly obtained from their friends. This result echoed previous research suggested that the external factors that lead to juvenile deliquency are friends, the use of leisure time, and the influence of the surrounding environment [18]. Further, one quantitative research showed that there is a significant positive correlation between peer conformity and deliquency tendency in adolescents [19].

Financial issues are one of internal factors that also hamper adolescents’ identity achievement in terms of life goals, education, and career. An investigation revealed that socio-economic conditions had a significant effect on students’ motivation to continue to higher education in high schools in Central Java, Indonesia [20]. In addition, another study showed that the socio-economic conditions of families are correlated with the increasing problem of juvenile delinquency in North Sumatera, Indonesia [21].

### Ex-localization as a stressor

The environment in which the adolescents lived was a stressor during their identity achievement and had psychological impacts on both the family and the adolescents. Such impacts included fear, worry, anxiety, and pity. Sitepu [11] lists that the negative impacts of localization, including disturbances to households and family life, personality issues, the spread of sexually transmitted diseases, and sexual crimes involving adolescents and the people around them. In addition to affecting the families, ex-localization also has other impacts on adolescents, such as a reluctance to interact socially (lack of socialization) and abusive and profane language. It was reported that teenagers in Gambilagu, Semarang, Indonesia must deal with fighting, having no interactions with other teenagers outside of the local area, and engaging in negative behaviors and activities [9].

### Families’ efforts to facilitate their adolescents during identity achievement

The families’ efforts included monitoring, advising, instilling religious values, and avoiding violence. Suyanto and Narwoko [22] assert that family has an important role in facilitating the identity achievement process. Monitoring here means that adolescents are given the freedom to engage in an activity they choose and mingle with anyone they like as long as they are responsible for their choices. This responsibility is applied to themselves as well as to the family. Such a condition is in line with one of the family developmental tasks noted by Friedman [23], that is, a family should offer a balance of freedom and responsibility since teenagers are young adults and must begin to exercise their own autonomy. Bronfenbrenner [7] argues that parental monitoring is a powerful tool to prevent risk-taking behaviors.

In addition to monitoring, other family efforts included advising for descent behaviors and avoiding doing violence upon the adolescents. Advising is a form of communication family provide to their children. A study conducted revealed that interpersonal communication between parents and adolescents is very important for avoiding premarital sex [6]. Teenagers who do not experience abuse within their families achieve better identity development than those who do [9].

The families also made efforts to instill religious values. It is argued that parents that actively promote their children’s religious identities are using a direct method, giving advice, and explaining the main points of the religion related to worshipping—and then inviting the adolescents to participate [24]. A study showed that a commitment to religion is negatively correlated with drug abuse in teenagers [25].

Hodge et al. [26] argues that adolescents’ participation in religious activities could prevent them from drinking alcohol, smoking marijuana, and other forms of drug abuse. Marcia [27] found that teenagers who are committed to a religion have an *achievement* identity status, or a moratorium identity [28].

### Family expectations for the future

While accompanying their adolescents in ex-localization, the participants expressed their furture expectations and wishes. These expectations were related to of having a desired teenager, and support from public figures as well as government.

The first expectation (i.e. Having a desired teenager) was the manifestation of the family educational function, where families have an obligation to teach and educate their children according to the children’s stage of development. Such a condition in line with Boobak, Lowdermilk, and Jensen [28], who state that the function of education is to teach skills, attitude, and knowledge related to the other functions. Thus, children are sent to school to receive an education, tailor their skills to their talents and interests, prepare them to face their future adulthood and fulfill their role adults, and educate them in accordance with their developmental level.

Furthermore, the families’ expectations are getting support from public figures and government. They stated that adolescents should have access to positive activities that involve public figures as good role models. Such activities could be started through optimization of existing youth organizations, including Qur’an recitation and religious education. Lastly, expectation to the government is related to the programs to serve the parents about developmental tasks in teenagers as well as health and social issues associated with living in prostitution areas.

## Conclusion

The results of this study highlight various information related to family facillitation of adolescents during identity achievement in ex-localization. There were five themes that emerged: 1) the identity achievement of adolescents living in ex-localization is similar to that of adolescents in general, 2) the domination of external barriers during identity achievement, 3) ex-localization as a stressor, 4) families’ efforts to facilitate their adolescents during identity achievement, and 5) family expectations for the future.

With regard to the theme of external barrier domination, the results show that the environment was very impactful to adolescent identity achievement. Family support is important in achieving this developmental task.

This study also identified differences in the families’ remarks: parents in ex-localization were more concerned with the changes occurring in female adolescents. This valuation was influenced by the perceptions, knowledge, and closeness of the families and the teenagers. Moreover, the psychological responses of the participants who have lived for a long time in ex-localization, those who are new to ex-localization, and those who used to directly participate in prostitution were similar. Families who lived in an environment far from the practice of prostitution expressed their feelings in a more emotional way than those lived nearby the prostitution sites.

### Recommendations

Families are expected to facilitate their adolescents during identity achievement by performing their roles correctly, exchanging ideas with other families that have adolescents, and visiting health services to obtain more information related to adolescent growth and development.

Mental health nurses, who work in public health service, could provide health education related to the growth, development, and self-identity developmental tasks of adolescents as well as offering psychosocial services, especially to prevent permanent psychological effects resulting from living in ex-localization. Health education and mentoring can be implemented by mental health nurses by stimulating the development of self-identity through the formation of therapeutic adolescent’s therapy group.

The government is expected to provide programs not only in the economic field and to monitor the spread of HIV but also to lighten the psychological burdens experienced by families.

## Data Availability

The datasets from this study is available from the corresponding author upon reasonable request.

## References

[1] Central Bureau Statistics (2015). Projection of Indonesian society. 2010–203.

[2] Central Bureau of Statistics. Population According to Age Group, Urban Area, and Gender. 2010. https://sp2010.bps.go.id/index.php/site/tabel?tid=263&wid=3500000000. Retrieved from https://sp2010.bps.go.id/index.php/site/tabel?tid=263&wid=3500000000. Downloaded on June 5 2017.

[3] Papalia DE, Olds SW, Feldman RD. Human development, (10th ed). Jakarta: Salemba Humanika; 2011.

[4] Mahlawi PN, Rachma N. Permasalahan remaja yang tinggal di area lokalisasi Gambilangu Semarang. In: Proceedings Seminar Nasional & Internasional; 2012.

[5] Purwadi P (2012). Peroses pembentukan identitas diri remaja. Humanitas (Jurnal Psikologi Indonesia).

[6] Putra NFP (2013). Peranan komunikasi interpersonal orang tua dan anak dalam mencegah perilaku seks pranikah di SMA Negeri 3 Samarinda kelas XII. Ejournal Ilmu Komunikasi.

[7] Bronfenbrenner U, Morris PA. The bioecological model of human development. Handbook of child psychology, 1. 2007. Retrieved from https://scholar.google.co.id/scholar?hl=id&as_sdt=0%2C5&q=Bronfenbrenner+%282007%29+&btnG=#d=gs_cit&u=%2Fscholar%3Fq%3Dinfo%3Amtz0Nw1KmCQJ%3Ascholar.google.com%2F%26output%3Dcite%26scirp%3D0%26hl%3Did. Downloaded on June 5 2017.

[8] Purwadi. Hubungan gaya pengasuhan orang tua dengan eksplorasi dan komitmen remaja dalam domain pekerjaan (Masters Thesis). Bandung: Universitas Padjadjaran; 2000.

[9] Idemudia ES, Makhubela S. Gender difference, exposure to domestic violence and adolescents' identity development. Gender Behav. 2011;9(1):3443-65. Retrived from https://scholar.google.co.id/scholar?hl=id&as_sdt=0%2C5&q=Idemudia%2C+E.+S.%2C+%26+Makhubela%2C+S.+%282011%29.+Gender+difference%2C+exposure+to+domestic+violence+and%EF%BB%BF+Adolescents%27+identity+development.&btnG=. Downloaded on June 5 2017.

[10] Chang L, Lansford JE, Schwartz D, Farver JM (2004). Marital quality, maternal depressed affect, harsh parenting, and child externalising in Hong Kong Chinese families. Int J Behav Dev.

[11] Sitepu A. Dampak lokalisasi prostitusi terhadap perilaku remaja di sekitarnya. 2009. Retrieved from https://www.researchgate.net/publication/42362599_Dampak_Lokalisasi_Prostitusi_Terhadap_Perilaku_Remaja_Di_Sekitarnya. Downloaded on June 5^th^ 2017.

[12] Polit DF, Beck CT (2012). Nursing research generating and assesing evidence *for nursing practice*.

[13] Clarke V, Braun V. Teaching thematic analysis: overcoming challenges and developing strategies for effective learning. The psychologist. 2013;26(2):120-3. Retrieved from http://eprints.uwe.ac.uk/21155/. Downloaded on November 5^th^ 2017.

[14] Brownlee S. Behavior can be baff ling when young minds are taking shape. US News and World Report. 1999:44–54. 10557497

[15] Spear LP (2000). The adolescent brain and age-related behavioral manifestations. Neurosci Biobehav Rev.

[16] Newman BM, Newman PR. Development through life: a psychosocial approach. Cengage Learning. 2017.

[17] Stuart GW. Prinsip dan praktik keperawatan kesehatan jiwa. (B. A. Keliat & J. Pasaribu, Trans.). Elsevier: Singapore; 2016. (Original work published 2013).

[18] Fuadi A. Esensi manusia dalam prespektif filsafat pendidikan islam. TARBIYAH. 2016;23(2).

[19] Saputro BM, Soeharto TNED. Hubungan antara konformitas terhadap teman sebaya dengan kecenderungan kenakalan pada remaja. Insight. 2012;10(1):1-15. Retrieved from https://scholar.google.co.id/scholar?q=hubungan-antara-konformitas-terhadap-teman-sebaya-dengan-kecenderungan-kenakalan-pada-remaja&hl=id&as_sdt=0&as_vis=1&oi=scholart. Downloaded on November 5^th^ 2017.

[20] Suryani N. Pengaruh Kondisi Sosial dan Ekonomi Orang Tua Terhadap Motivasi Melanjutkan Pendidikan ke Perguruan Tinggi. Dinamika Pendidikan. 2006;1(2). Retrieved from https://journal.unnes.ac.id/nju/index.php/DP/article/view/476. Downloaded on November 5^th^ 2017.

[21] Barus CP. Sosial Ekonomi Keluarga dan Hubungannya dengan Kenakalan Remaja di Desa Lantasan Baru Kecamatan Patumbak Kabupaten Deli Serdang. Welfare StatE. 2013;2(1). Retrieved from https://jurnal.usu.ac.id/index.php/ws/article/view/2138. Downloaded on November 5th 2017.

[22] Suyanto B, Narwoko JD (2004). Sosiologi Teks Pengantar dan Terapan.

[23] Friedman MM, Bowden VR, Jones EG (2010). Buku Ajar Keperawatan Keluarga: Riset, teori, dan praktik Edisi 5.

[24] Wahyuningsih H. Peran Orangtua dalam Pembentukan Identitas Agama (Religious Identity Formation) Remaja. Indigenous: Jurnal Ilmiah Psikologi. 2009;11(1).

[25] Mason WA, Windle M (2001). Family, religious, school and peer influences on adolescent alcohol use: a longitudinal study. J Stud Alcohol.

[26] Hodge DR, Cardenes P, Montoya H (2001). Substance use: spirituality and religious participation as protective factors among Rurals youths. Soc Work Res.

[27] Santrock JW (2012). Adolescence.

[28] Bobak L, Jensen (2004). Buku ajar keperawatan maternitas.

